# Extra corporal membrane oxygenation in general thoracic surgery: a new single veno-venous cannulation

**DOI:** 10.1186/1749-8090-6-52

**Published:** 2011-04-14

**Authors:** Redha Souilamas, Jihane I Souilamas, Khalid Alkhamees, Jean-Pierre Hubsch, Jean-Claude Boucherie, Reem Kanaan, Yves Ollivier, Mauricio Sauesserig

**Affiliations:** 1 Thoracic surgery department, European Georges Pompidou Hospital, 20 rue Leblanc 75015 Paris, France; 2 Paris Descartes Medical School University, Rue de l'École de Médecine, 75006 Paris France; 3 Intensive care unit and anesthesia department, European Georges Pompidou Hospital, 20 rue Leblanc 75015 Paris, France; 4 Chest medicine department, Cochin Hospital, Rue Saint Jacques, 75006 Paris, France; 5 Cardiovascular surgery department, European Georges Pompidou Hospital, 20 rue Leblanc 75015 Paris, France

## Abstract

Extracorporeal membrane oxygenation (ECMO) is used in severe respiratory failure to maintain adequate gas exchange. So far, this technique has not been commonly used in general thoracic surgery. We present a case using ECMO for peri-operative airway management for pulmonary resection, using a novel single-site, internal jugular, veno-venous ECMO cannula.

## Clinical summary

**Figure 1:**
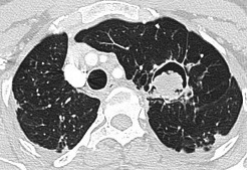
Patient Computer Tomography Scan (CT scan) left lung aspergilloma which requires resection.

The patient was a 45-year-old woman with aspergilloma in the left upper lung (figure 1) and recurrent haemoptysis. Several arteriographies with embolizations had been completed with no long-lasting success. Segmentectomy was then discussed. Her forced expiratory volume in 1 second (FEV_1_) predicted was 42% and her left pulmonary perfusion was 75%. These results made surgery unlikely and risky for 2 reasons: the absence of left lung ventilation required during surgery and the potential risk of postoperative respiratory insufficiency.

Lung transplantation was discussed but the patient was not listed because she did not have respiratory end-stage disease. A multidisciplinary decision was made to proceed with pulmonary resection under peri-operative VV-ECMO support with the Avalon cannula. This strategy made it possible to cease lung ventilation during surgery and created an option to bridge the patient to transplantation in case of postoperative respiratory failure. Pre-lung transplant evaluation was carried out before surgery.

**Figure 2:**
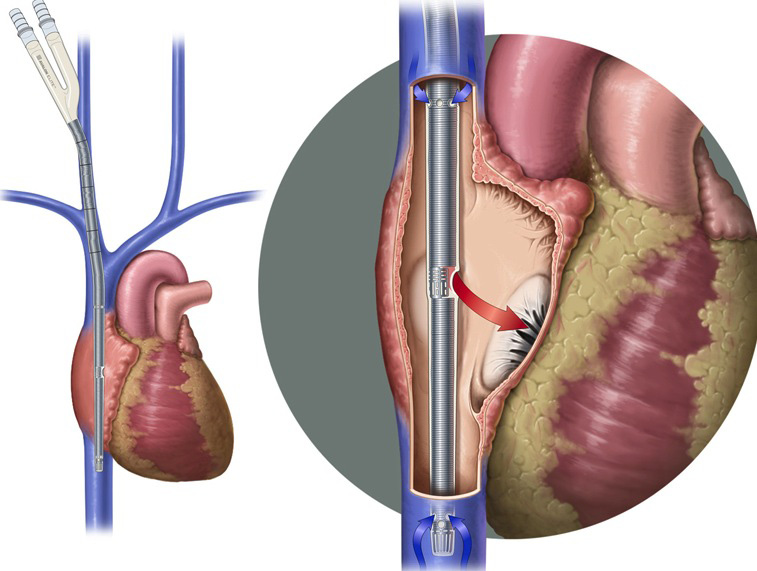
Avallon cannula description.

**Figure 3:**
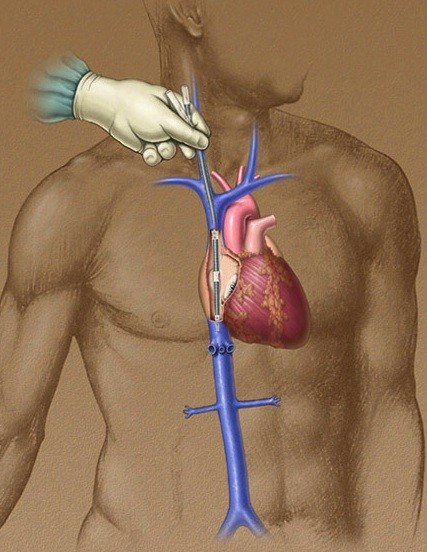
**Right internal Jugular cannulation description**.

The peri-operative management of such airway compromise is difficult but critical. A 23F Avalon cannula (Figure 2) (Avalon Laboratories, LLC, Rancho Dominguez, Calif) was inserted into the right internal jugular vein after puncture and dilatation under general anesthesia (figure 3), using transesophageal echocardiographic guidance.

The double-lumen jugular Avalon catheter (23F) was connected to a heparin-coated ECMO circuit consisting of a rotary pump and a polymethylpentene oxygenator. A 3.0 liters/min blood flow was easily achieved allowing sufficient O_2_and CO_2_transfers. The patient underwent uneventful segmentectomy and was extubated at the end of surgery. ECMO support weaned off after achieving satisfactory gas exchange 12 hours post-operatively. The 10 months follow up was satisfactory. FEV_1 _predicted was 38%. without recurrence of haemoptysis.

## Comments

ECMO support is increasingly being utilised in the management of severe respiratory failure [1] as a bridge to lung transplantation [2] and in management of post-transplant primary graft dysfunction [3]. VV ECMO usually requires a dual site implantation. This new single-site cannulation technique through internal jugular vein brings obvious benefits with the poster-lateral thoracotomy position and allows the maintenance of adequate gas exchange during surgery.

Two thoracic surgical cases have been reported using dual-site bilateral femoral VV ECMO. The first one was for curative surgery in a patient with papillary thyroid carcinoma invading the trachea [4]. The second for carinal resection and reconstruction after left pneumonectomy [5].

To our knowledge, this innovative technique of cannulation has been reported in lung transplantation [2,3], but never reported in general thoracic surgery. Despite its potential risks [6], such a cannulation remains an effective alternative airway management option in patients with a significant lung function insufficiency who require pulmonary resection. Furthermore, the use of ECMO support even in thoracic surgery should be limited to institutions that have multi-disciplinary cardiac and vascular department with extensive knowledge in ECMO technology and the management of complications.

## Consent

Written informed consent was obtained from the patient for publication of this case report and accompanying images. A copy of the written consent is available for review by the Editor-in-Chief of this journal.

## Competing interests

The authors declare that they have no competing interests.

## Authors' contributions

RS conceived, supervise, wrote the article.

JS participated in its design, writing process and bibliography

MS, KA, participated in its coordination and correction on the surgical part.

RK participated in its coordination on the pre-operative part

YO, JPH, JCB conceived participated in its coordination on the anesthesiologic and extracorporal assistance part.

All authors read and approved the final manuscript.
